# Kinetics of Glycoxidation of Bovine Serum Albumin by Glucose, Fructose and Ribose and Its Prevention by Food Components

**DOI:** 10.3390/molecules191118828

**Published:** 2014-11-17

**Authors:** Izabela Sadowska-Bartosz, Sabina Galiniak, Grzegorz Bartosz

**Affiliations:** 1 Department of Biochemistry and Cell Biology, University of Rzeszów, Zelwerowicza 4, 35-601 Rzeszów, Poland; 2 Department of Molecular Biophysics, University of Łódź, Pomorska 141/143, 90-236 Łódź, Poland

**Keywords:** glycation, kinetics, glucose, fructose, ribose, polyphenols, flavonoids, AGEs

## Abstract

The aim of this study was to compare the kinetics of the glycoxidation of bovine serum albumin (BSA) as a model protein by three sugars: glucose, fructose and ribose, using fluorometric measurements of the content of advanced glycation end products (AGEs), protein-bound fructosamine, dityrosine, *N*'-formylkynurenine, kynurenine, tryptophan, the content of advanced oxidation protein products (AOPP), protein carbonyl groups, as well as thiol groups. Moreover, the levels of glycoalbumin and AGEs were determined by using an enzyme-linked immunosorbent assay. Based on the kinetic results, the optimal incubation time for studies of the modification of the glycoxidation rate by additives was chosen, and the effects of 25 compounds of natural origin on the glycoxidation of BSA induced by various sugars were examined. The same compounds were found to have different effects on glycoxidation induced by various sugars, which suggests caution in extrapolation from experiments based on one sugar to other sugars. From among the compounds tested, the most effective inhibitors of glycoxidation were: polyphenols, pyridoxine and 1-cyano-4-hydroxycinnamic acid.

## 1. Introduction

In the body, proteins are subject to a variety of enzymatic and non-enzymatic modifications. One of the unavoidable consequences of metabolism is the non-enzymatic reaction of proteins with reducing sugars (glycation or Maillard reaction). Protein glycation is initiated by a nucleophilic addition reaction between the free amino group from a protein, lipid or nucleic acid and the carbonyl group of reducing saccharides. This reaction forms a reversible Schiff base, which rearranges over a period of days to produce ketoamine or Amadori products. The Amadori products undergo dehydration and rearrangements followed by other reactions, such as cyclization, oxidation and dehydration, to form more stable advanced glycation end products (AGEs) [1]. AGE formation takes place under normal physiologic conditions, but is accelerated in hyperglycemia [2,3]. Glycation alters the structure and functional properties of proteins, which adversely affects cellular metabolism. The accumulation of glycation products is observed in human and animal tissues during aging and is associated with various diseases, including, first of all, diabetes and diabetic nephropathy, microangiopathy and atherosclerosis [4,5]. The serum levels of AGEs have been found to be elevated also in cystic fibrosis [6] and neurodegenerative diseases, such as Alzheimer’s disease, Parkinson’s disease [7], Creutzfeldt–Jacob disease [8], as well as amyotrophic lateral sclerosis [9]. Glycation has been well-studied for many proteins, both short- (e.g., human hemoglobin) and long-lived (such as collagen and lens crystallin). It should be mentioned that many studies have been done on bovine serum albumin (BSA), which has high (76%) sequence homology to human serum albumin [10].

The main glycating sugar, present in the body at the highest concentration, is d-glucose. However, d-fructose is more reactive, which leads to enhanced glycation in fructosemia [11]. Fructose is a common monosaccharide that is found naturally in its free form in honey, fruits and other plant material and in a bound form as a component of sucrose. When taken orally, fructose is absorbed in the small intestine and efficiently adsorbed in the liver; as a consequence, there is no big postprandial increase in the blood fructose concentration. Diabetic patients have elevated serum and urinary fructose levels. Serum fructose concentrations in patients with diabetes are 12.0 ± 3.8 μM, while in healthy subjects, values of 8.1 ± 1.0 μM are found (*p* < 0.001) [12].

d-ribose is a pentose occurring in every cell type and is a component of such important molecules as riboflavin, adenine nucleotides and ribonucleic acids. Ribose has the ability to react with proteins to produce glycated derivatives. The body contains about 16 mg of ribose per liter of blood (~100 μM) [13]; another study reported the concentration of free ribose in human blood plasma to be 7 μM (0–17 μM) [14]. Ribose (0.01–0.1 mM) is present also in the cerebrospinal fluid [13].

Glycation can be catalyzed by metals and is usually associated with the generation of reactive oxygen species (ROS) and oxidation; the combination of both processes is often referred to as glycoxidation.

The possibility of reducing glycation and tissue AGEs is an approachable target for delaying or preventing the onset of diabetic complications. Although synthetic compounds are powerful drugs that inhibit AGEs formation or break cross-links, they can also have severe side effects. For example, aminoguanidine and other hydrazine drugs, such as hydralazine, isoniazid and gentamicins, are either toxic or show adverse side effects, because of the depletion of essential carbonyls, such as pyridoxal phosphate (vitamin B_6_) [15]. Therefore, it is critical to develop effective and safe agents to protect diabetic individuals from complications [16]. The treatment of diabetes mellitus will benefit from on-going screening and development of novel compounds that offer combined antioxidant and anti-glycation properties [17]. Such studies are being performed employing conditions close to physiological ones [18,19]. Prevention of glycoxidation is also important for the food industry to avoid the formation of AGEs in food processing [20].

The aim of this paper was to compare the effect of chosen compounds of natural origin, in particular polyphenols, on the glycoxidation of BSA by three different sugars. In preliminary experiments, the kinetics of glycoxidation was studied in order to find optimal conditions for the examination of the effects of potential inhibitors on the rate of glycation. The conditions of the experiments were far from physiological, both in terms of BSA concentration, which was lower by an order of magnitude than that in the blood plasma, and especially in terms of sugar concentrations, which were at least two orders of magnitude higher than the physiological ones. However, as the amount of product formed in a chemical reaction is an integral of the rate constant and concentrations of substrates over time, increased substrate concentrations allow for obtaining appropriate amounts of a product in a shorter time. Thus, a several-day incubation provides amounts of glycoxidation products equivalent to those formed over several weeks or months *in vivo*. However, extrapolation to the *in vivo* situation may not be straightforward, due to the homeostatic systems of the body. Nevertheless, results obtained in model experiments permit preliminary screening for potential inhibitors of glycoxidation.

## 2. Results

### 2.1. Kinetics of Glycoxidation

**Figure 1:**
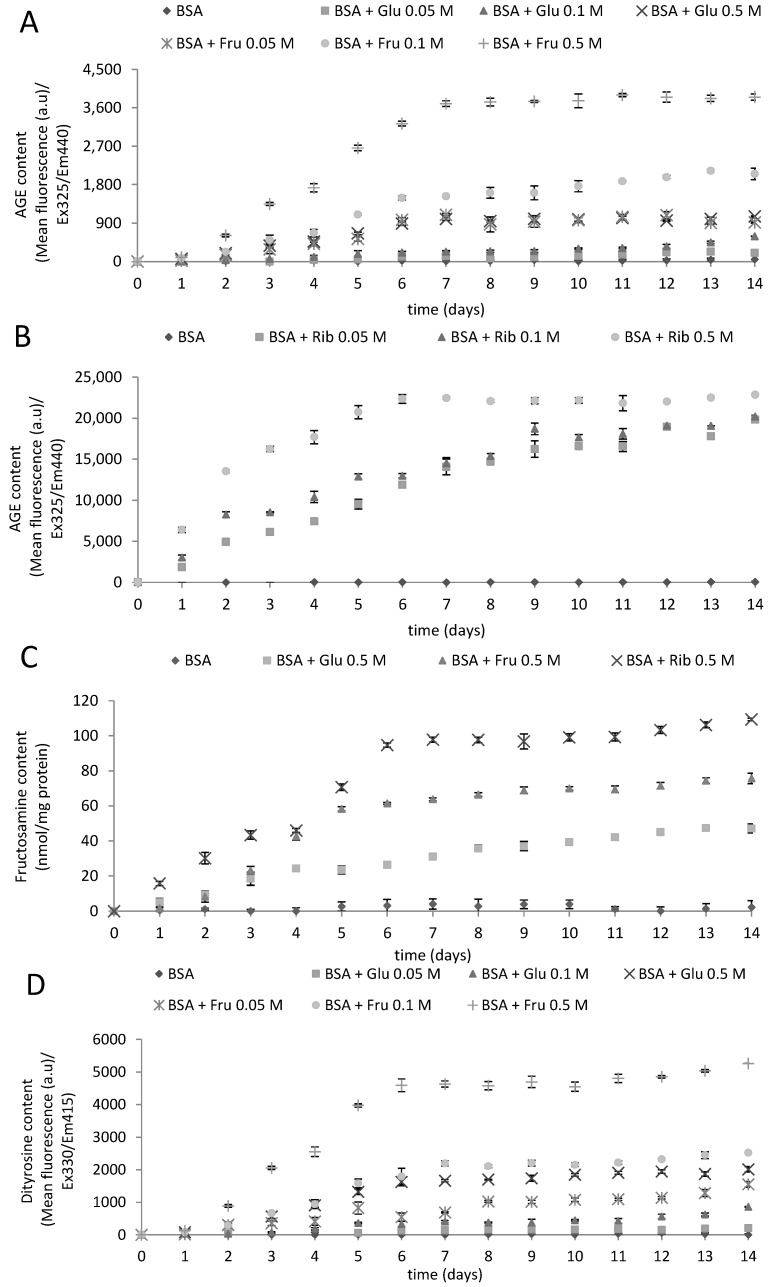

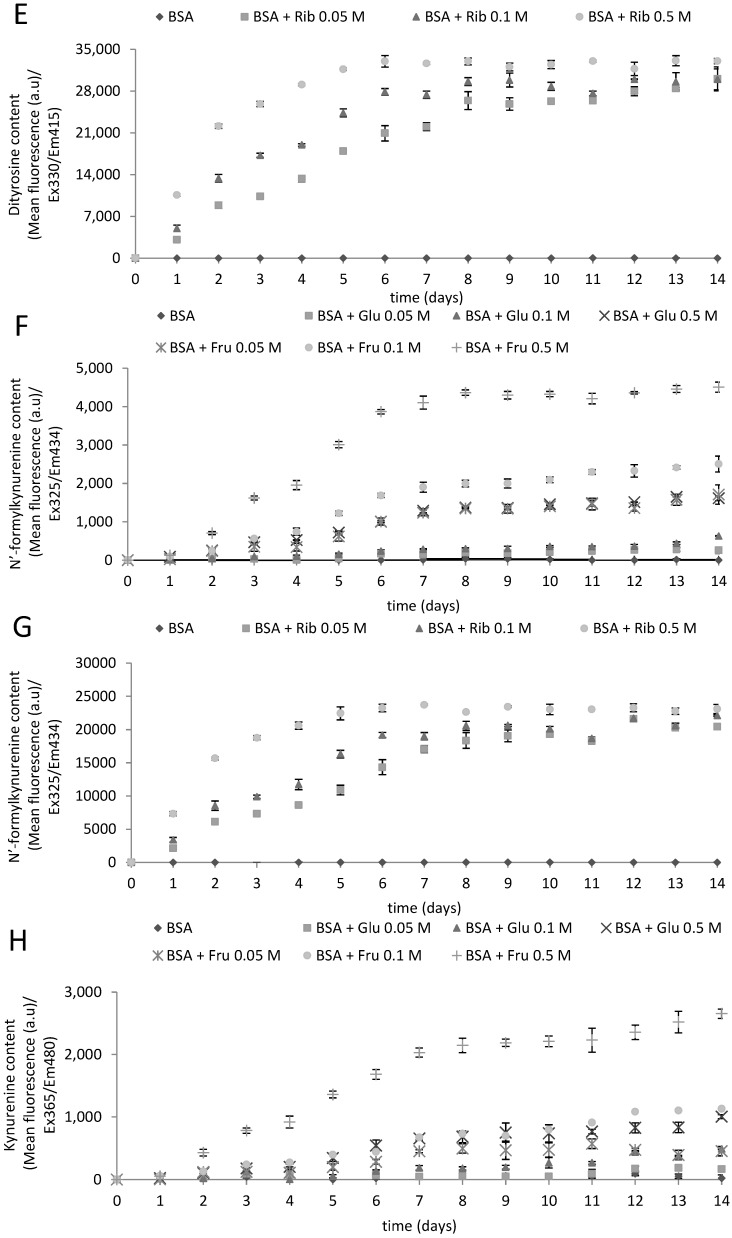

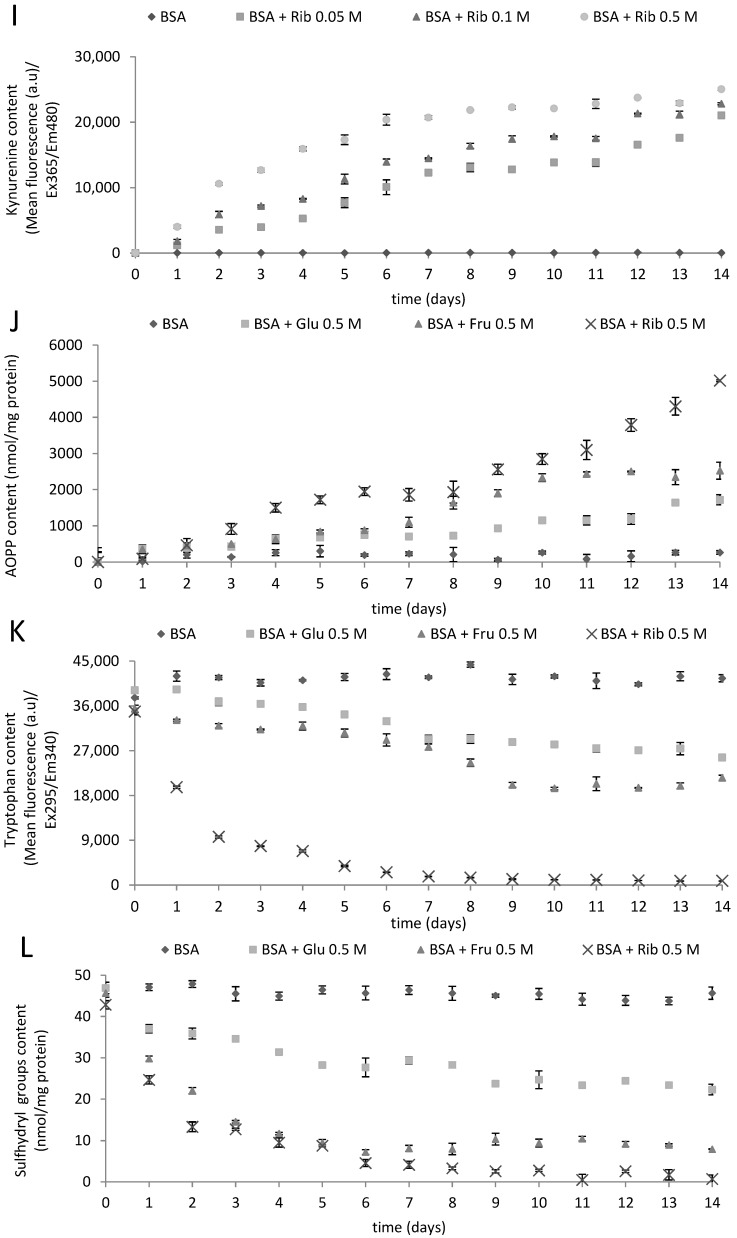
The time course of the glycoxidation of BSA incubated at 37 °C for 14 days with 0.05, 0.1 and 0.5 M glucose (Glu), fructose (Fru) and ribose (Rib): AGE fluorescence (**A**,**B**); fructosamine content (**C**); dityrosine content (**D**,**E**); *N*'-formylkynurenine content (**F**,**G**); N-formylkynurenine content (**H**,**I**); advanced oxidation protein products (AOPP) content (**J**); tryptophan fluorescence; (**K**) and sulfhydryl group content (**L**).

We monitored changes in the values of chosen parameters describing protein glycoxidation and oxidative modifications during a 14-d incubation with reducing sugars. The level of AGEs, determined fluorometrically, increased with time during incubation with the sugars, until reaching a plateau level (Figure 1A,B). The behavior of fructosamine followed the same pattern, although the higher ribose concentration interfered with the assay (Figure 1C), due to the reaction with nitroblue tetrazolium.

The time-course of parameters reflecting protein oxidative damage, such as the levels of dityrosine (Figure 1D,E), *N*'-formylkynurenine (Figure 1F,G), kynurenine (Figure 1H,I) and advanced oxidation protein products (AOPP; Figure 1J) showed a similar behavior, increasing in parallel with those of AGEs and fructosamine. Tryptophan fluorescence (Figure 1K) and sulfhydryl group content (Figure 1L) decreased in parallel. Thus, the glycation of BSA was accompanied by protein oxidation, in full agreement with the idea of glycoxidation.

### 2.2. Protection against Glycoxidation

In search for compounds that could effectively inhibit glycoxidation, we have compared the effects of three standard inhibitors of glycation, six amino acids or their derivatives and peptides, two reference antioxidants, three iron chelators, six organic acids, two polyamines and 10 naturally-occurring polyphenols. BSA was incubated in the absence and in the presence of the additives for six days, and the extent of glycoxidation was estimated on the basis of changes in the fluorometric indices of glycoxidation (the content of AGE, dityrosine, *N*'-formylkynurenine and kynurenine) and AOPP content.

Aminoguanidine and metformin are two standard inhibitors of glycoxidation applied in *in vivo* experiments [5]. In our case, metformin slightly enhanced the rate of glycoxidation by glucose, did not affect significantly the glycoxidation by ribose and had a moderate inhibitory effect on the glycoxidation by fructose in this simple *in vitro* system. In contrast, aminoguanidine significantly inhibited glycoxidation by glucose and fructose and moderately inhibited glycoxidation by ribose. Another inhibitor of glycoxidation, pyridoxine, was the most effective inhibitor of glycoxidation by all three sugars. The fluorescence measured after six days for samples incubated with pyridoxine was in most cases lower than at Day 0, apparently due to the consumption of fluorophores, contributing to the fluorescence in the reactions with this compound; the same effect was noted in some other cases (Table 1, Table 2 and Table 3).

**Table 1:** The effect of various additives (1 mM) on the extent of the glycoxidation of BSA induced by glucose (0.5 M), estimated with fluorometric parameters. **^Δ^** *p* < 0.05, **^#^** *p* < 0.01, * *p* < 0.001 (paired Student’s *t*-test). Results significantly different from samples incubated with the sugar without additives are shown in bold. DETAPA, diethylenetriaminepentaacetic acid; NTA, nitrilotriacetic acid.

Additive	AGE	Dityrosine	*N*'-formylkynurenine	Kynurenine	AOPP
BSA, no sugar	3.55 ± 5.16 *	3.46 ± 5.61 *	1.57 ± 5.37 *	7.27 ± 10.06 *	**17.25 ± 6.58 ^#^**
BSA + glucose, no additive	100	100	100	100	100
DMSO	103.78 ± 10.32	100.56 ± 10.59	103.45 ± 10.91	**232.47 ± 23.09 ^#^**	91.11 ± 9.34
*Standard Antiglycating Agents*					
Aminoguanidine	41.91 ± 2.69 *	29.39 ± 4.13 *	38.07 ± 3.07 *	**66.58 ± 10.87 ^Δ^**	**41.31 ± 4.87 ^#^**
Metformin	**112.75 ± 3.34 ^Δ^**	**116.00 ± 2.79 ^#^**	**124.59 ± 3.01 ^#^**	**119.83 ± 6.21 ^Δ^**	118.21 ± 9.02
Pyridoxine	<0 *	<0 *	<0 *	**193.42 ± 14.04 ^#^**	6.19 ± 4.27 *
*Antioxidants*					
Captopril	**34.52 ± 7.55 ^#^**	**31.85 ± 6.81 ^#^**	**37.62 ± 7.22 ^#^**	87.27 ± 16.74	**33.66 ± 6.14 ^#^**
Tiron	**31.3 ± 10.73 ^#^**	**28.91 ± 10.92 ^#^**	**33.12 ± 11.23 ^#^**	**72.55 ± 15.79 ^Δ^**	65.87 ± 13.88
*Metal Chelators*					
EDTA	98.14 ± 2.27	92.47 ± 3.40	99.53 ± 4.81	87.01 ± 7.95	106.87 ± 13.37
DETAPA	**135.79 ± 2.61 ^#^**	**127.51 ± 1.63 ^Δ^**	**125.27 ± 4.11 ^Δ^**	94.94 ± 2.24	107.18 ± 17.16
NTA	**124.59 ± 9.51 ^Δ^**	113.80 ± 8.24	112.30 ± 8.92	94.03 ± 13.82	109.73 ± 8.69
*Amino Acids and Derivatives, Peptides*					
Arginine	120.14 ± 9.56	106.81 ± 9.16	108.58 ± 8.69	104.33 ± 14.37	107.86 ± 14.18
Carnosine	**122.00 ± 6.13 ^Δ^**	**120.20 ± 6.46 ^Δ^**	**135.06 ± 7.29 ^#^**	**152.47 ± 9.75 ^#^**	127.47 ± 31.73
Cysteamine	**20.19 ± 9.35 ^#^**	**19.16 ± 9.82 ^#^**	**22.82 ± 10.75 ^#^**	**48.48 ± 22.52 ^Δ^**	**46.15 ± 10.14 ^Δ^**
Glutathione oxidized	**136.72 ± 10.56 ^Δ^**	**140.81 ± 11.13 ^Δ^**	**144.95 ± 11.84 ^Δ^**	149.44 ± 20.76	110.25 ± 8.37
Glutathione reduced	**51.49 ± 13.28 ^Δ^**	**49.26 ± 10.97 ^#^**	**49.61 ± 10.87 ^#^**	111.08 ± 25.92	83.66 ± 8.70
Glycine	140.89 ± 20.65	143.49 ± 18.41	148.09 ± 21.45	148.40 ± 28.52	110.04 ± 10.32
*Organic Acids*					
1-Cyano-4-hydroxycinnamic acid	4.89 ± 1.60 *	4.59 ± 1.26 *	6.12 ± 2.29 *	19.48 ± 5.36 *	**31.62 ± 9.55 ^#^**
4-Hydroxy cinnamic acid	94.70 ± 7.05	97.77 ± 6.09	104.29 ± 6.23	92.12 ± 12.43	89.07 ± 9.50
Lipoic acid	**89.23 ± 2.41 ^Δ^**	**89.35 ± 2.42 ^#^**	97.02 ± 3.17	97.73 ± 2.80	91.71 ± 9.89
*Para*-aminobenzoic acid	**152.04 ± 18.58 ^Δ^**	**150.09 ± 16.01 ^Δ^**	**163.83 ± 17.49 ^Δ^**	**149.39 ± 15.80 ^Δ^**	129.74 ± 25.42
Pyruvic acid	**82.29 ± 3.11 ^#^**	**84.56 ± 2.68 ^#^**	**91.26 ± 2.88 ^Δ^**	**73.94 ± 6.16 ^#^**	94.54 ± 2.27
Quinic acid	**72.93 ± 8.44 ^Δ^**	**75.19 ± 8.59 ^Δ^**	**79.49 ± 8.34 ^Δ^**	**56.54 ± 12.28 ^Δ^**	88.53 ± 11.99
*Organic Polybases*					
Spermidine	**143.88 ± 4.11 ^#^**	**137.43 ± 3.23 ^#^**	151.7 ± 3.39 *	233.07 ± 8.83 *	117.47 ± 16.58
Spermine	144.22 ± 19.98	139.62 ± 23.29	153.53 ± 22.79	**195.06 ± 35.58 ^Δ^**	118.37 ± 13.14
*Polyphenols*					
Caffeic acid	<0 *	<0 *	<0 *	**180.71 ± 9.74 ^#^**	**43.38 ± 14.08 ^Δ^**
Ellagic acid	96.47 ± 6.16	**58.82 ± 4.37 ^#^**	92.01 ± 6.56	107.71 ± 35.95	103.99 ± 41.12
Ferulic acid	<0 *	<0 *	**29.34 ± 7.35 ^#^**	**156.28 ± 19.39 ^Δ^**	**53.27 ± 9.22 ^Δ^**
Gallic acid	460.03 ± 9.73 *	**249.02 ± 14.12 ^#^**	432.07 ± 11.04 *	**586.37 ± 49.71 ^#^**	615.09 ± 24.62 *
Genistein	27.46 ± 0.97 *	23.86 ± 0.79 *	27.77 ± 1.43 *	46.85 ± 1.77 *	**44.43 ± 10.14 ^Δ^**
Kaempferol	20.77 ± 2.09 *	13.98 ± 1.58 *	20.18 ± 2.43 *	22.12 ± 4.38 *	**45.97 ± 18.44 ^Δ^**
Naringin	28.90 ± 0.44 *	22.05 ± 0.17 *	24.38 ± 0.35 *	103.39 ± 4.68	**35.87 ± 11.61 ^Δ^**
Propyl gallate	<0 *	<0 *	<0 *	198.18 ± 5.78 *	71.84 ± 20.08
Quercitrin	3.37 ± 0.38 *	1.11 ± 0.28 *	1.01 ± 0.17 *	3.31 ± 0.58 *	**47.35 ± 14.16 ^Δ^**
Rutin	2.94 ± 0.35 *	1.15 ± 0.34 *	1.26 ± 0.23 *	2.79 ± 0.14 *	**42.26 ± 5.76 ^#^**

Out of two amino acids studied, arginine moderately decreased glycoxidation by fructose and ribose, having a slight, although not significant, stimulatory effect on the glycoxidation by glucose, while glycine slightly enhanced glycoxidation by glucose and fructose and had no discernible effect on the glycoxidation by ribose. Cysteamine and reduced glutathione inhibited glycoxidation by all sugars; interestingly, oxidized glutathione provided also some protection against glycoxidation by glucose and ribose, while enhancing glycoxidation induced by fructose.

Numerous papers suggested that glycoxidation can be inhibited by antioxidants and metal chelators [5,21,22]. Both model antioxidants used, captopril and tiron, significantly inhibited the glycoxidation by glucose and fructose. Captopril inhibited the glycoxidation by fructose, while the effect of tiron was very low, except for AOPP. Glycoxidation by ribose was not affected considerably by either antioxidant; the changes were concordant for all parameters measured.

**Table 2:** The effect of various additives (1 mM) on the extent of the glycoxidation of BSA induced by fructose (0.5 M), estimated with fluorometric parameters. **^Δ^** *p* < 0.05, **^#^** *p* < 0.01, * *p* < 0.001 (paired Student’s *t*-test).

Additive	AGE	Dityrosine	*N*'-formylkynurenine	Kynurenine	AOPP
BSA, no sugar	0.67 ± 0.97 *	0.65 ± 1.05 *	0.29 ± 0.99 *	1.52 ± 2.10 *	8.91 ± 3.40 *
BSA + fructose, no additive	100	100	100	100	100
DMSO	**68.54 ± 4.64 ^#^**	**62.86 ± 3.89 ^#^**	**67.52 ± 4.32 ^#^**	**134.00 ± 10.30 ^Δ^**	**70.55 ± 3.61 ^#^**
*Standard Antiglycating Agents*					
Aminoguanidine	19.53 ± 1.58 *	17.76 ± 0.667 *	19.20 ± 0.75 *	39.31 ± 1.96 *	**44.15 ± 3.33 ^#^**
Metformin	**82.45 ± 5.02 ^Δ^**	**83.67 ± 5.14 ^Δ^**	**87.36 ± 4.91 ^Δ^**	**75.52 ± 7.14 ^Δ^**	123.16 ± 13.28
Pyridoxine	<0 *	<0 *	<0 *	181.88 ± 3.75 *	**34.26 ± 4.68 ^#^**
*Antioxidants*					
Captopril	27.87 ± 0.78 *	25.15 ± 0.78 *	28.50 ± 0.93 *	59.26 ± 2.32 *	16.28 ± 3.17 *
Tiron	**97.32 ± 0.70 ^Δ^**	**104.07 ± 0.78 ^#^**	**103.62 ± 0.98 ^Δ^**	**105.68 ± 1.72 ^Δ^**	**44.98 ± 4.78 ^#^**
*Metal Chelators*					
EDTA	98.57 ± 2.99	97.09 ± 2.99	99.2 ± 1.50	99.76 ± 2.01	111.12 ± 12.93
DETAPA	**63.07 ± 3.04 ^#^**	**59.98 ± 2.92 ^#^**	**58.47 ± 2.79 ^#^**	**49.35 ± 7.83 ^#^**	**87.21 ± 4.25 ^Δ^**
NTA	**107.64 ± 3.03 ^Δ^**	102.55 ± 3.12	101.09 ± 2.80	**93.19 ± 4.03 ^Δ^**	109.27 ± 5.26
*Amino Acids and Derivatives, Peptides*					
Arginine	**93.26 ± 3.56 ^Δ^**	**87.62 ± 3.70 ^Δ^**	**89.68 ± 4.06 ^Δ^**	**81.59 ± 4.85 ^Δ^**	107.2 ± 41.66
Carnosine	97.12 ± 5.10	96.28 ± 4.74	103.05 ± 5.13	104.80 ± 8.01	109.53 ± 18.10
Cysteamine	54.44 ± 1.83 *	53.36 ± 1.78 *	56.81 ± 2.18 *	**71.95 ± 4.41 ^#^**	**46.10 ± 13.99 ^Δ^**
Glutathione oxidized	**111.61 ± 1.64 ^#^**	**113.09 ± 2.61 ^#^**	**115.42 ± 2.35 ^#^**	**114.77 ± 3.19 ^#^**	120.90 ± 16.07
Glutathione reduced	**78.10 ± 7.50 ^Δ^**	**78.39 ± 6.58 ^Δ^**	**77.68 ± 6.80 ^Δ^**	**69.38 ± 8.54 ^Δ^**	**129.12 ± 2.89 ^#^**
Glycine	**118.53 ± 7.88 ^Δ^**	**118.7 ± 7.03 ^Δ^**	**120.96 ± 6.82 ^Δ^**	**120.01 ± 8.89 ^Δ^**	102.15 ± 21.96
*Organic Acids*					
1-Cyano-4-hydroxycinnamic acid	5.57 ± 0.16 *	5.14 ± 0.13 *	6.27 ± 0.16 *	20.81 ± 0.59 *	**52.02 ± 13.55 ^Δ^**
4-Hydroxycinnamic acid	**88.85 ± 3.17 ^Δ^**	**90.26 ± 3.39 ^Δ^**	94.86 ± 3.67	93.19 ± 5.23	63.30 ± 17.30
Lipoic acid	**132.99 ± 4.67 ^#^**	**130.7 ± 4.22 ^#^**	**137.82 ± 4.29 ^#^**	**129.87 ± 4.37 ^#^**	**150.17 ± 8.03 ^#^**
*Para*-aminobenzoic acid	102.75 ± 4.31	97.82 ± 3.74	106.23 ± 4.13	107.31 ± 4.52	111.79 ± 9.52
Pyruvic acid	**61.70 ± 7.64 ^#^**	**62.90 ± 7.79 ^#^**	**65.43 ± 8.03 ^#^**	**39.36 ± 9.49 ^#^**	87.62 ± 5.22
Quinic acid	96.86 ± 2.00	98.96 ± 1.73	**104.02 ± 1.80 ^Δ^**	98.33 ± 2.61	85.14 ± 6.43
*Organic Polybases*					
Spermidine	101.10 ± 1.64	94.96 ± 3.30	102.77 ± 2.56	**153.21 ± 8.47 ^#^**	93.39 ± 12.04
Spermine	**120.74 ± 7.01 ^Δ^**	**118.59 ± 7.42 ^Δ^**	**125.58 ± 7.11 ^Δ^**	**150.61 ± 5.94 ^#^**	98.82 ± 49.59
*Polyphenols*					
Caffeic acid	<0 *	<0 *	<0 *	**239.70 ± 68.36 ^Δ^**	**79.04 ± 6.87 ^Δ^**
Ellagic acid	31.62 ± 4.73 *	21.03 ± 3.68 *	29.80 ± 4.41 *	**60.44 ± 7.81 ^#^**	**129.61 ± 5.19 ^Δ^**
Ferulic acid	<0 *	<0 *	0.42 ± 3.25 *	92.74 ± 5.87	**43.52 ± 3.32 ^#^**
Gallic acid	59.54 ± 1.65 *	33.05 ± 1.20 *	55.43 ± 1.18 *	217.27 ± 2.94 *	92.99 ± 21.89
Genistein	14.88 ± 0.61 *	12.64 ± 0.34 *	14.97 ± 0.49 *	20.47 ± 0.25 *	**69.88 ± 10.88 ^Δ^**
Kaempferol	14.36 ± 1.15 *	9.98 ± 0.77 *	13.63 ± 1.10 *	18.55 ± 2.11 *	**64.21 ± 7.72 ^Δ^**
Naringin	27.03 ± 0.30 *	21.53 ± 0.38 *	24.90 ± 0.97 *	99.74 ± 3.01	**76.34 ± 4.21 ^Δ^**
Propyl gallate	<0 *	<0 *	<0 *	**54.36 ± 6.62 ^#^**	**41.08 ± 11.75 ^Δ^**
Quercitrin	2.62 ± 0.26 *	1.77 ± 0.22 *	2.08 ± 0.30 *	1.84 ± 0.23 *	43.63 ± 2.57 *
Rutin	2.48 ± 0.11 *	1.82 ± 0.07 *	2.20 ± 0.10 *	1.82 ± 0.09 *	**50.28 ± 7.56 ^#^**

**Table 3:** The effect of various additives (1 mM) on the extent of the glycoxidation of BSA induced by ribose (0.5 M), estimated with fluorometric parameters. **^Δ^** *p* < 0.05, **^#^** *p* < 0.01, * *p* < 0.001 (paired Student’s *t*-test).

Additive	AGE	Dityrosine	*N*'-formylkynurenine	Kynurenine	AOPP
BSA, no sugar	0.67 ± 0.97 *	0.65 ± 1.05 *	0.29 ± 0.99 *	1.52 ± 2.10 *	1.82 ± 0.70 *
BSA + ribose, no additive	100	100	100	100	100
DMSO	**92.02 ± 0.64 ^#^**	**92.38 ± 0.67 ^#^**	**93.92 ± 0.84 ^#^**	113.33 ± 0.96 *	**109.68 ± 0.87 ^#^**
*Standard Antiglycating Agents*					
Aminoguanidine	78.07 ± 1.53 *	70.13 ± 0.32 *	79.71 ± 0.44 *	80.21 ± 2.06 *	**64.35 ± 4.39 ^#^**
Metformin	99.66 ± 1.77	100.52 ± 1.67	**105.57 ± 1.63 ^Δ^**	91.19 ± 3.96	**80.84 ± 4.72 ^Δ^**
Pyridoxine	<0 *	<0 *	<0 *	95.09 ± 2.42	10.02 ± 3.45 *
*Antioxidants*					
Captopril	93.66 ± 0.41 *	**94.57 ± 0.80 ^#^**	**98.39 ± 0.69 ^Δ^**	102.39 ± 2.97	**91.08 ± 2.02 ^Δ^**
Tiron	**97.32 ± 0.70 ^Δ^**	**104.07 ± 0.78 ^#^**	**103.62 ± 0.98 ^Δ^**	**105.68 ± 1.72 ^Δ^**	**51.85 ± 4.39 ^#^**
*Metal Chelators*					
EDTA	98.67 ± 0.59	97.64 ± 6.72	99.29 ± 7.77	100.31 ± 8.44	96.71 ± 0.74
DETAPA	**104.09 ± 0.48 ^#^**	99.73 ± 0.62	100.59 ± 0.78	978.65 ± 1.79	**76.42 ± 3.41 ^#^**
NTA	111.73 ± 0.79 *	**107.94 ± 1.07 ^#^**	**107.87 ± 0.80 ^#^**	104.63 ± 2.52	**86.01 ± 2.59 ^Δ^**
*Amino Acids and Derivatives, Peptides*					
Arginine	**89.83 ± 0.86 ^#^**	89.06 ± 0.80 *	87.61 ± 0.96 *	97.67 ± 1.91	**86.78 ± 4.06 ^Δ^**
Carnosine	93.56 ± 0.43 *	93.68 ± 0.35 *	**99.28 ± 0.24 ^Δ^**	102.90 ± 0.77	**77.96 ± 3.48 ^#^**
Cysteamine	**78.55 ± 1.87 ^#^**	**79.47 ± 1.85 ^#^**	**82.13 ± 1.98 ^#^**	104.22 ± 3.52	**93.21 ± 1.68 ^Δ^**
Glutathione oxidized	**83.63 ± 4.11 ^#^**	**91.42 ± 3.07 ^Δ^**	**87.73 ± 4.38 ^Δ^**	106.09 ± 4.06	95.55 ± 3.30
Glutathione reduced	**85.58 ± 1.45 ^#^**	**91.99 ± 1.15 ^#^**	**86.17 ± 1.16 ^#^**	103.70 ± 3.42	109.26 ± 3.92
Glycine	99.46 ± 0.55	103.24 ± 0.24 *	**104.19 ± 0.99 ^#^**	**109.24 ± 2.23 ^#^**	**89.25 ± 3.25 ^Δ^**
*Organic Acids*					
1-Cyano-4-hydroxycinnamic acid	18.29 ± 0.11 *	16.46 ± 0.04 *	18.54 ± 0.05 *	35.31 ± 0.19 *	**33.38 ± 5.90 ^#^**
4-Hydroxycinnamic acid	**102.44 ± 0.28 ^#^**	**104.63 ± 0.40 ^#^**	110.93 ± 0.26 *	**97.48 ± 0.27 ^#^**	96.52 ± 3.52
Lipoic acid	**105.45 ± 0.61 ^#^**	**102.81 ± 0.83 ^Δ^**	108.73 ± 0.35 *	**111.00 ± 2.01 ^#^**	**83.08 ± 5.73 ^Δ^**
*Para*-aminobenzoic acid	85.53 ± 1.05 *	80.87 ± 1.05 *	87.46 ± 0.93 *	99.43 ± 1.78	**82.13 ± 3.72 ^Δ^**
Pyruvate	104.70 ± 2.98	105.50 ± 3.08	**111.00 ± 3.13 ^Δ^**	99.18 ± 4.47	98.48 ± 8.42
Quinic acid	**108.40 ± 0.90 ^#^**	**110.98 ± 0.90 ^#^**	116.92 ± 1.11 *	**107.96 ± 0.80 ^#^**	105.43 ± 3.09
*Organic Polybases*					
Spermidine	38.84 ± 0.37 *	36.07 ± 0.21 *	40.85 ± 0.39 *	87.18 ± 0.65 *	**44.69 ± 5.23 ^#^**
Spermine	32.06 ± 1.86 *	29.54 ± 1.80 *	33.71 ± 1.87 *	71.84 ± 0.59 *	**67.50 ± 3.97 ^#^**
*Polyphenols*					
Caffeic acid	**45.63 ± 22.36 ^Δ^**	**56.37 ± 24.57 ^Δ^**	**48.62 ± 12.47 ^#^**	96.67 ± 6.67	64.26 ± 0.88 *
Ellagic acid	**74.18 ± 2.54 ^#^**	**74.19 ± 2.95 ^#^**	**74.74 ± 2.85 ^#^**	44.32 ± 1.84 *	**77.97 ± 6.66 ^Δ^**
Ferulic acid	14.04 ± 0.52 *	28.05 ± 0.49 *	18.08 ± 0.51 *	99.22 ± 1.05	41.60 ± 2.21 *
Gallic acid	73.48 ± 1.87 *	**86.01 ± 1.52 ^#^**	79.09 ± 1.49 *	64.21 ± 1.38 *	**81.57 ± 3.22 ^Δ^**
Genistein	29.40 ± 0.99 *	28.48 ± 1.02 *	30.75 ± 0.93 *	33.91 ± 0.82 *	17.42 ± 3.80 *
Kaempferol	29.23 ± 1.95 *	24.84 ± 1.96 *	29.68 ± 1.88 *	19.98 ± 1.81 *	84.23 ± 6.49
Naringin	53.60 ± 1.21 *	50.86 ± 1.11 *	53.28 ± 1.20 *	68.38 ± 0.70 *	**58.63 ± 4.45 ^#^**
Propyl gallate	66.71 ± 1.60 *	55.39 ± 2.09 *	65.65 ± 1.77 *	81.82 ± 1.30 *	**55.23 ± 3.53 ^#^**
Quercitrin	14.20 ± 0.15 *	12.13 ± 0.13 *	14.19 ± 0.18 *	8.37 ± 0.07 *	**50.17 ± 3.76 ^#^**
Rutin	15.79 ± 0.30 *	13.40 ± 0.35 *	15.78 ± 0.35 *	7.88 ± 0.14 *	**64.22 ± 6.26 ^Δ^**

We checked the effects of chelators, ethylenediaminetetraacetate acid (EDTA), diethylenetriaminepentaacetic acid (DETAPA) and nitrilotriacetic acid (NTA) on BSA glycoxidation. Judging from the changes in AGE, dityrosine and N-formylkynurenine fluorescence, EDTA had no significant effect on glycoxidation induced by any sugar; DETAPA significantly inhibited only fructation; while NTA had no significant effect on the glycoxidation induced by glucose or fructose and slightly increasing the glycoxidation by ribose. Changes in AOPP content followed the same pattern with the only exception of a small decrease of this parameter by DETAPA and NTA in the case of ribose and by DETAPA in the case of fructose. These data point to considerable differences in the effects of the same substances on glycoxidation induced by different sugars, which were visible also with further substances studied.

Lipoic acid moderately inhibited glycoxidation by glucose, having no significant effect on the glycoxidation induced by ribose and promoting to some extent glycoxidation by fructose and ribose, though decreasing the AOPP level evoked by ribose. *Para*-aminobenzoic acid enhanced glycoxidation by glucose, slightly inhibited glycoxidation by ribose and did not affect the glycoxidation by fructose. Quinic acid inhibited to some extent glycoxidation by glucose, did not affect the glycoxidation by fructose and somewhat enhanced glycoxidation by ribose. 4-Hydroxycinnamic acid did not affect glycoxidation by glucose, slightly inhibited glycoxidation by fructose and somewhat enhanced glycoxidation by ribose, while 1-cyano-4-hydroxycinnamic acid was an efficient inhibitor of glycoxidation induced by all sugars studied.

The naturally occurring polyamines, spermine and spermidine, significantly inhibited glycoxidation by ribose, did not affect or slightly enhanced (spermine) glycoxidation by fructose and promoted glycoxidation by glucose.

The natural polyphenols used, with the exception of gallic acid and ellagic acid in the case of glucose-induced glycoxidation, were good inhibitors of glycoxidation induced by all three sugars.

The effects measured were always consistent for three parameters: the level of AGEs and the content of dityrosine and *N*'-formylkynurenine. The results for the *N*'-formylkynurenine fluorescence were sometimes inconsistent, apparently due to the interference of the substances added with that of formylkynurenine. The results obtained for AOPP were generally consistent with those obtained for AGEs, and the content of dityrosine and *N*'-formylkynurenine, with a few exceptions (pyruvate for glycoxidation by glucose; ellagic acid, reduced glutathione, metformin and DETAPA for fructation and NTA in the case of glycoxidation by ribose), where the opposite direction of significant changes was obtained for the fluorescence of AGEs, dityrosine and *N*'-formylkynurenine, on the one hand, and changes in AOPP, on the other hand.

In order to check the validity of the protective effects of the additives on the glycoxidation of BSA, we estimated also the level of carbonyl groups by a fluorometric assay and the level of glycated albumin by the Bovine Glycated Albumin ELISA Kit. The results generally confirm those obtained by fluorometric and AOPP assays. Aminoguanidine and pyridoxine decreased carbonyl group formation induced by all sugars, while metformin only that induced by glucose (slightly). Captopril and tiron decreased BSA carbonylation induced by glucose and fructose, while NTA only that induced by glucose. Cysteamine, carnosine, reduced glutathione, 1-cyano-4-hydroxycinnamic acid and pyruvic acid attenuated the formation of carbonyl groups induced by all sugars, oxidized glutathione that by glucose and glycine that by glucose and fructose. *Para*-aminobenzoic acid slightly enhanced carbonylation induced by ribose. Spermidine enhanced carbonylation induced by fructose, while decreasing that induced by ribose. Most polyphenols (caffeic acid, ferulic acid, genistein, naringin, propyl gallate and rutin) inhibited carbonylation induced by all three sugars, ellagic acid that induced by glucose and fructose, while gallic acid enhanced carbonylation induced by glucose, while attenuating that induced by fructose and ribose (Table 4).

**Table 4:** The effect of various additives (1 mM) on the protein carbonyl content of glycated BSA induced by glucose, fructose and ribose (0.5 M), estimated with the protein carbonyl fluorometric assay. **^Δ^** *p* < 0.05, **^#^** *p* < 0.01, * *p* < 0.001 (paired Student’s *t*-test).

Additive	Glucose	Fructose	Ribose
BSA + sugar, no additive	100	100	100
DMSO	101.31 ± 2.37	99.90 ± 2.09	101.58 ± 1.89
*Standard Antiglycating Agents*			
Aminoguanidine	74.41 ± 0.44 *	70.63 ± 1.683 *	88.98 ± 1.00 *
Metformin	**96.57 ± 0.46 ^#^**	97.74 ± 2.836	99.35 ± 0.54
Pyridoxine	43.35 ± 2.55 *	43.08 ± 0.16 *	71.85 ± 1.86 *
*Antioxidants*			
Captopril	84.93 ± 1.02 *	86.56 ± 1.46 *	98.45 ± 1.92
Tiron	83.10 ± 0.76 *	**94.41 ± 0.86 ^#^**	98.14 ± 1.73
*Metal Chelators*			
NTA	**95.35 ± 1.61 ^Δ^**	97.78 ± 0.51	100.31 ± 0.71
*Amino Acids and Derivatives, Peptides*			
Arginine	97.16 ± 5.84	98.35 ± 3.39	**98.79 ± 0.58 ^Δ^**
Carnosine	**93.19 ± 2.04 ^#^**	**92.55 ± 1.84 ^#^**	**95.36 ± 1.44 ^#^**
Cysteamine	82.25 ± 0.94 *	86.00 ± 0.84 *	88.43 ± 1.93 *
Glutathione oxidized	**95.69 ± 0.69 ^#^**	99.24 ± 1.27	100.73 ± 0.67
Glutathione reduced	**88.75 ± 2.77 ^#^**	**93.19 ± 3.52 ^Δ^**	**96.71 ± 0.93 ^Δ^**
Glycine	**95.61 ± 1.16 ^#^**	99.62 ± 1.35	**97.51 ± 1.22 ^Δ^**
*Organic Acids*			
1-Cyano-4-hydroxycinnamic acid	66.41 ± 2.25 *	72.08 ± 1.89 *	89.86 ± 0.61 *
4-Hydroxycinnamic acid	100.64 ± 3.46	97.73 ± 0.98	101.17 ± 3.11
Lipoic acid	100.51 ± 8.54	**96.19 ± 1.23 ^Δ^**	100.78 ± 3.55
*Para*-aminobenzoic acid	102.88 ± 2.92	93.71 ± 4.11	105.51 ± 0.58 *
Pyruvic acid	84.34 ± 1.06 *	87.07 ± 0.27 *	**98.94 ± 0.50 ^Δ^**
Quinic acid	97.05 ± 3.35	99.05 ± 2.67	98.35 ± 1.87
*Organic Polybases*			
Spermidine	**103.5 ± 0.65 ^Δ^**	96.48 ± 2.82	92.4 ± 0.20 *
*Polyphenols*			
Caffeic acid	46.8 ± 1.39 *	61.52 ± 1.31 *	86.04 ± 0.42 *
Ellagic acid	**94.53 ± 0.31 ^#^**	**89.76 ± 2.97 ^#^**	100.05 ± 0.55
Ferulic acid	56.83 ± 3.53 *	60.96 ± 0.87 *	85.12 ± 2.72 *
Gallic acid	**115.89 ± 4.67 ^#^**	**94.30 ± 1.88 ^Δ^**	**95.66 ± 1.55 ^#^**
Genistein	61.43 ± 2.95 *	66.39 ± 0.23 *	86.23 ± 1.10 *
Naringin	57.83 ± 4.10 *	69.61 ± 1.03 *	92.52 ± 0.42 *
Propyl gallate	57.38 ± 1.53 *	63.13 ± 1.83 *	83.97 ± 1.15 *
Rutin	62.11 ± 3.98 *	67.99 ± 0.64 *	77.25 ± 0.61 *

The level of glycated BSA estimated by ELISA was reduced by aminoguanidine and pyridoxine in the case of glycoxidation induced by all three sugars and by metformin in the case of glycoxidation induced by ribose. Tiron was protective against glycation induced by all sugars, while captopril against that induced by glucose and fructose. NTA slightly enhanced glycation by glucose. Arginine decreased glycation induced by ribose, carnosine that by fructose. Cysteine and reduced glutathione attenuated glycation by fructose and ribose, while glycine that induced by all sugars. Oxidized glutathione slightly enhanced glycation by glucose and ribose. 1-Cyano-4-hydroxycinnamic acid attenuated glycation induced by all sugars. Lipoic acid and quinic acid slightly protected against glycation by glucose. *Para*-aminobenzoic acid slightly protected against glycation by fructose and somewhat increased that by glucose and ribose. Pyruvic acid inhibited fructose-induced glycation, but slightly increased that by ribose. Spermidine was protective against ribose-induced glycation. Most polyphenols (caffeic acid, ferulic acid, genistein, naringin, propyl gallate and rutin) protected against glycation induced by all sugars; gallic acid enhanced glycation by glucose and attenuated that by ribose, while ellagic acid slightly enhanced glycation by glucose and decreased that by fructose (Table 5).

**Table 5:** The effect of various additives (1 mM) on the glycated albumin content in BSA incubated with glucose, fructose and ribose (0.5 M), estimated with the Bovine Glycated Albumin ELISA Kit. The levels of glycated albumin in samples incubated with sugars only are assumed as 100%; **^Δ^** *p* < 0.05, **^#^** *p* < 0.01, * *p* < 0.001 (paired Student’s *t*-test).

Additive	Glucose	Fructose	Ribose
BSA + sugar, no additive	100	100	100
DMSO	**98.68 ± 0.76 ^Δ^**	99.18 ± 2.79	99.03 ± 0.87
*Standard Antiglycating Agents*			
Aminoguanidine	75.38 ± 0.31 *	79.34 ± 0.85 *	**79.1 ± 4.69 ^#^**
Metformin	100.01 ± 2.20	**98.58 ± 0.32 ^Δ^**	99.39 ± 2.77
Pyridoxine	53.24 ± 0.69 *	72.82 ± 1.18 *	74.48 ± 2.69 *
*Antioxidants*			
Captopril	88.08 ± 0.06 *	89.35 ± 2.01 *	98.06 ± 5.92
Tiron	94.79 ± 0.48 *	**95.00 ± 2.73 ^Δ^**	**95.44 ± 2.47 ^Δ^**
*Metal Chelators*			
NTA	101.66 ± 0.10 *	100.86 ± 0.69	105.87 ± 2.26
*Amino Acids and Derivatives, Peptides*			
Arginine	100.65 ± 2.44	97.34 ± 2.49	**95.61 ± 0.72 ^#^**
Carnosine	101.20 ± 3.82	87.54 ± 0.41 *	92.33 ± 4.76
Cysteamine	**93.64 ± 1.32 ^#^**	**94.56 ± 1.41 ^#^**	92.99 ± 8.31
Glutathione oxidized	**101.24 ± 0.43 ^Δ^**	99.79 ± 2.77	**102.68 ± 1.00 ^Δ^**
Glutathione reduced	98.65 ± 2.79	**96.47 ± 1.39 ^Δ^**	**91.23 ± 3.57 ^Δ^**
Glycine	97.25 ± 0.01 *	**96.67 ± 1.15 ^Δ^**	**98.33 ± 3.85 ^Δ^**
*Organic Acids*			
1-Cyano-4-hydroxycinnamic acid	86.38 ± 0.27 *	82.21 ± 0.46 *	82.88 ± 2.30 *
4-Hydroxycinnamic acid	102.02 ± 2.58	102.53 ± 1.71	97.06 ± 5.98
Lipoic acid	98.40 ± 0.12 *	97.9 ± 3.49	98.98 ± 0.04
*Para*-aminobenzoic acid	**104.78 ± 1.51 ^#^**	**97.53 ± 1.20 ^Δ^**	**102.57 ± 0.59 ^Δ^**
Pyruvic acid	98.20 ± 1.71	91.35 ± 0.87 *	**102.62 ± 0.69 ^Δ^**
Quinic acid	**97.65 ± 0.63 ^#^**	98.50 ± 2.12	97.62 ± 2.64
*Organic Polybases*			
Spermidine	99.73 ± 1.04	97.32 ± 3.51	**93.11 ± 0.53 ^#^**
*Polyphenols*			
Caffeic acid	50.43 ± 0.19 *	66.40 ± 1.82 *	79.48 ± 0.99 *
Ellagic acid	**101.48 ± 0.472 ^#^**	**94.17 ± 2.664 ^Δ^**	101.90 ± 0.74
Ferulic acid	59.00 ± 0.16 *	62.31 ± 2.63 *	81.26 ± 1.52 *
Gallic acid	116.07 ± 0.13 *	100.52 ± 0.46	**92.50 ± 4.35 ^Δ^**
Genistein	77.97 ± 1.69 *	86.8 ± 0.28 *	84.08 ± 1.92 *
Naringin	78.20 ± 0.06 *	72.53 ± 2.96 *	91.43 ± 0.11 *
Propyl gallate	53.15 ± 0.20 *	65.76 ± 1.01 *	73.83 ± 1.42 *
Rutin	69.32 ± 0.30 *	70.28 ± 0.18 *	**79.61 ± 4.06 ^#^**

**Table 6:** Correlation coefficients between various parameters of glycoxidation and protein oxidative damage for BSA subject to glycation with various sugars in the presence of eight polyphenols. E, ELISA; F, fluorometry, S, spectrophotometry; **^∆^** *p* < 0.05, **^#^** *p* < 0.01, * *p* < 0.005.

Parameters of Glycoxidation	AGEs (E)	AGEs (F)	Dityrosine (F)	*N*'-formylkynurenine (F)	Kynurenine (F)	Protein Carbonyls (F)
**Glucose**						
AGEs (F)	0.926 *					
Dityrosine (F)	0.963 *	0.962 *				
*N*'-formylkynurenine (F)	0.450	0.598	0.636			
Kynurenine (F)	0.000	−0.037	−0.074	0.090		
AOPP (S)	0.107	−0.037	0.074	0.378	0.750	
Carbonyl protein (F)	**0.857 ^∆^**	**0.815 ^∆^**	**0.852 ^∆^**	0.504	−0.143	
Glycated albumin (E)	**0.857 ^∆^**	0.963 *	**0.926 ^#^**	0.738	−0.071	**0.821 ^∆^**
**Fructose**						
AGEs (F)	**0.926 ^#^**					
Dityrosine (F)	**0.926 ^#^**	1.000 *				
*N*'-formylkynurenine (F)	**0.901 ^#^**	0.972 *	0.972 *			
Kynurenine (F)	−0.107	0.074	0.074	0.036		
AOPP (S)	0.393	0.630	0.630	0.559	0.679	
Carbonyl protein (F)	0.964 *	**0.926 ^#^**	**0.926 ^#^**	**0.847 ^∆^**	0.000	
Glycated albumin (E)	**0.786 ^∆^**	**0.926 ^#^**	**0.926 ^#^**	**0.847 ^∆^**	0.074	**0.857 ^∆^**
**Ribose**						
AGEs (F)	**0.786 ^∆^**					
Dityrosine (F)	0.536	**0.857 ^∆^**				
*N*'-formylkynurenine (F)	0.714	0.964 *	**0.893 ^#^**			
Kynurenine (F)	−0.143	−0.071	0.250	0.143		
AOPP (S)	0.679	0.500	0.571	0.393	−0.107	
Carbonyl protein (F)	0.357	0.536	0.607	0.607	−0.036	
Glycated albumin (E)	0.321	0.214	0.143	0.250	−0.321	**0.821 ^∆^**

### 2.3. Validity of Fluorometric and Spectrophotometric Measures of Glycoxidation

In order to check the validity of the results based on the assay of fluorometric and spectrophotometric indices of glycoxidation and protein oxidative damage, the correlation was evaluated between the AGE content evaluated by ELISA and fluorometric parameters (AGE content evaluated fluorometrically, dityrosine, *N*'-formylkynurenine and kynurenine content), AOPP content evaluated spectrophotometrically, carbonyl group content estimated by a fluorometric test and the level of glucose-, fructose- and ribose-glycated BSA estimated by ELISA. The correlations were evaluated for BSA incubated with various sugars in the presence of various flavonoids (rutin, naringin, gallic acid, propyl gallate, caffeic acid, genistein, ferulic acid and ellagic acid). These substances may interfere with spectroscopic/fluorometric measurements, thus giving less reliable data. The results summarized in Table 6 point to a good correlation between the AGE content evaluated by ELISA and the content of glycated albumin, carbonyl groups and AGE and dityrosine content evaluated fluorometrically. In the case of glycoxidation induced by fructose, the AGE content estimated by ELISA correlated well with glycated albumin, carbonyl groups, AGE, dityrosine and N'-formylkynurenine content evaluated fluorometrically. For glycoxidation induced by ribose, the level of AGEs determined by ELISA showed a statistically significant correlation only with the level of AGEs determined fluorometrically.

## 3. Discussion

We studied the kinetics and protection against the glycoxidation of albumin, a model protein often used in studies of this type [23]. Albumin is the main protein of blood plasma and has a wide variety of physiological and pharmacological functions, including the maintenance of oncotic pressure, binding and transport of fatty acids, bilirubin, metal ions, nitric oxide and drugs. It contributes also significantly to the antioxidant capacity of blood plasma [3]. *In vivo*, the proportion of glycated albumin in healthy persons is in the range of 1%–10%; this proportion may increase two- to three-fold in diabetes mellitus [24] and is used as a short-to-intermediate-term marker for glycemic control in diabetes [23]. Therefore, there are good reasons for the use of this protein as a model to study the reactions of glycoxidation.

BSA is a 66.7-kDa protein rich in lysine (59; 10.1%) and arginine residues (23; 3.9%). *In vivo*, albumin is glycated at the arginine, lysine and cysteine residues. The structural modifications of albumin induced by glycoxidation include an increase in molecular weight and a higher exposure of hydrophobic sites to the solvent [3]. We found that standard indices of protein oxidation (formation of dityrosine, kynurenine, *N*'-formylkynurenine, AOPP, protein carbonyls and a decrease of tryptophan fluorescence and thiol group content) change in parallel to indices of BSA glycation (levels of AGE and fructosamine). Thus, * in vitro* glycoxidation of BSA under the atmosphere of air is associated with oxidation, which corresponds to the idea of glycoxidation and justifies the use of indices of protein oxidation to monitor the progress of glycoxidation. Moreover, we looked for a correlation between AGE content determined by ELISA and the levels of AGEs, dityrosine, *N*'-formylkynurenine and kynurenine determined fluorometrically, as well as AOPP level (estimated spectrophotometrically). The strong correlation between changes in the values of glycophore, dityrosine and *N*'-formylkynurenine suggests that either these modifications are formed in strict parallel in the system used or that their emission spectra overlap to some extent. Further studies are needed to distinguish between these possibilities. The correlations between the ELISA results and other parameters were lower for glycoxidation induced by fructose and not significant statistically for glycoxidation induced by ribose. These results do not necessarily discredit the application of fluorometric/spectrophotometric parameters for the estimation of glycoxidation induced by sugars other than glucose, especially ribose. Rather, it may be due to the fact that the antibodies used are directed against AGEs induced by glucose and may have lower reactivity for AGEs induced by other sugars.

Comparison of the time course of glycoxidation confirmed the sequence of the rate of glycoxidation, ribose > fructose > glucose, found also by other authors [25]. Suarez *et al*., found that the rate of glycoxidation by fructose (fructation) was about 10-times higher than that upon glycoxidation by glucose (glucation) [26]. It was also reported that with ribose (ribosylation) gives rise to advanced glycoxidation end products (AGEs) more rapidly than glycoxidation with glucose [27]. Comparison of the initial rates of glycophore formation from the data presented in Figure 1 indicates that this rate is 2.7- and 14.2-times, respectively, higher for fructose and ribose than for glucose. Assuming that the physiological concentrations of glucose, fructose and ribose are 5 mM, 8 μM [12] and 7 μM [14], the relative rates of fructosylation and ribosylation would be about 0.4% and 2.0%, respectively, of that of glucosylation. Thus, ribosylation may contribute significantly to the total glycation of blood plasma protein, especially if the level of ribose is increased.

d-ribose-induced glycoxidation of BSA results in rapid misfolding and formation of amyloid-like aggregates, which are cytotoxic to neural cells [28]. The role of d-ribose in glycoxidation and cross-linking of collagen has been investigated in *in vitro* studies of the triggering of skin ageing [29]. Luciano Viviani and colleagues prepared proteins glycated with ribose in a study of AGEs and their effects on pancreatic islet beta-cells. Results showed reduced cellular proliferation with a corresponding increase in the cell necrosis and cell apoptosis rate in comparison with untreated cells after five days of exposure to glycoxidation [30].

For studies of the effects of additives on glycoxidation, an incubation time of six days was chosen. This time corresponds to the end of the period of the time-dependent increase in glycoxidation before reaching a plateau. In our opinion, longer incubation times should not be used if the effect of inhibitors on the rate of glycoxidation is studied, as they may blur the inhibitory effects, allowing for reaching the plateau of glycoxidation-induced protein modifications in the presence of inhibitors, albeit after a longer incubation time. The results of the present study report mainly the effects of various substances on the rate of glycoxidation.

The effects of additives were often different for glycoxidation induced by various sugars. Apparently, this is due to different mechanisms of glycoxidation involving various intermediates. Irrespective of the mechanism, this result is important, perhaps providing an explanation of the divergent effects found by researchers studying glycoxidation induced by various sugars and suggesting care in the extrapolation of the results from one glycoxidation model to another.

The results of glycoxidation inhibition by various compounds showed the consistent behavior of the three fluorometric parameters of protein glycoxidation studied (the content of AGEs, dityrosine and *N*'-formylkynurenine), while the content of formylkynurenine is much less reliable and should not be recommended in studies of such a type. Furthermore, the level of AOPP is less convenient for the measurement of the extent of glycoxidation in the presence of additives for two reasons: (i) the higher variation of the results; and (ii) interference with the additives, which is the apparent reason for the differences in the results with respect to the fluorometric parameters for some compounds.

The study confirmed the glycoxidation-inhibitory action of pyridoxine and aminoguanidine [5]. Metformin and lipoic acid, known to inhibit glycoxidation *in vivo,* were ineffective in the *in vitro* system used [31]; apparently, these compounds require metabolic activation (reduction in the case of lipoic acid) and/or exert indirect effects *in vivo*.

The results confirm the inhibition of glycoxidation by natural polyphenols and indicate that their effect is universal, irrespective of which sugar is inducing glycoxidation (though the extent of inhibition varies between various sugars). Polyphenols, especially flavonoids, have been demonstrated to be effective inhibitors of glycoxidation. Previously, the inhibition of glycoxidation has been demonstrated for various polyphenols, including quercetin, genistein, tannic acid and gallic acid [16,32,33,34,35]. Although the concentration of flavonoids used in this study was high (1 mM), these compounds were also effective at lower concentrations (not shown); moreover, the sugar concentrations used in this study were unphysiologically high, so lower concentrations of protective compounds may be affective with respect to physiological glycoxidation. Anyhow, the effects of inhibitors of glycoxidation should be ascertained under conditions closer to physiological ones [18,19].

Flavonoids bind to albumin (and other proteins) [36], and this binding may enhance their protective efficiency against glycoxidation. As standard antioxidants had a stronger effect than metal chelators, it seems that the inhibitory effects of polyphenols were mainly due to their antioxidant than metal-chelating properties. The results of this study suggest that consumption of a polyphenol-rich diet may attenuate protein glycation to some extent, and the addition of polyphenols can be useful in reducing undesired glycoxidation in food processing.

Of interest is also the finding that 1-cyano-4-hydroxycinnamic acid is also a strong inhibitor of glycoxidation. This compound is known mainly as an inhibitor of monocarboxylate transporters [37], so it cannot be employed *in vivo*; however, the effect observed may point to a new class of potential inhibitors of glycoxidation.

## 4. Experimental Section

### 4.1. Reagents

All basic reagents were from Sigma-Aldrich (Poznan, Poland), unless indicated otherwise. Genistein, 4-hydroxycinnamic acid, naringin and quinic acid were purchased from Santa Cruz Biotechnology, Inc. (Santa Cruz, CA, United States). BSA (purity of 96%) was dissolved in 0.1 M sodium phosphate buffer, pH 7.4, at a concentration of 0.1 mM.

### 4.2. Glycation Conditions

Three sugars (glucose, fructose and ribose) were used as glycating agents at concentrations of 0.05, 0.1 and 0.5 M. The incubation mixtures contained BSA at a final concentration of 90 μM and 1 mM sodium azide as a preservative. The samples were incubated in closed vials at a temperature of 37 °C for 14 days. Samples were withdrawn, and the parameters of glycoxidation were measured every day.

### 4.3. Fluorometric Estimates of Glycoxidation

Fluorescence measurements were done by applying 200 µL of the sample on the plate. Fluorescence was measured at a wavelength of 325/440 nm (AGEs), 330/415 nm (dityrosine), 325/494 nm (*N*'-formylkynurenine), 365/480 nm (kynurenine) and 295/340 nm (tryptophan) [38].

### 4.4. Amadori Product

The formation of Amadori products was assessed using the method of Johnson and Baker [39], with nitroblue tetrazolium (NBT). One hundred microliters of the sample per well were transferred to a 96-well plate. One hundred microliters of NBT reagent (250 μM NBT in 0.1 M carbonate buffer, pH 10.35) were added to each well, and the plate was incubated at 37 °C for 2 h. Absorbance was measured at 525 nm. An absorption coefﬁcient of 12,640 cm^−1^ mol^−1^ for monoformazan [40] was used.

### 4.5. AOPP and Thiol Assay

AOPP concentration was estimated according to Witko-Sarsat *et al.* [41] by applying 50 µL of the sample and 150 μL of 0.1 M sodium phosphate buffer, pH 7.4, to microplate wells and adding 10 µL of 1.16 M sodium iodide and 20 µL of concentrated acetic acid. The absorbance was measured immediately at a wavelength of 340 nm. AOPP content was converted to nmol/mg protein. Thiol groups were estimated according to Ellman [42].

### 4.6. *ELISA*-Based AGEs, Carbonyl Groups and Bovine Glycated Albumin Assays

AGEs were also assayed with the ELISA Kit for AGEs from USCN Life Science Inc. (Product No. CEB353Ge, Wuhan, China), according to the instruction of the manufacturer. The carbonyl groups were determined by a fluorometric assay (OxiSelect™ Protein Carbonyl Fluorometric Assay, Cell Biolabs, Inc., San Diego, CA, USA) and the level of glucose-, fructose- and ribose-glycated albumin by the Bovine Glycated Albumin ELISA Kit (Shanghai Crystal day Biotech Co., Shanghai, China).

### 4.7. Prevention of Glycoxidation

For measurements of the effects of other compounds on the process of glycoxidation, BSA (90 μM) was incubated with 0.5 M sugars, azide and the additives (1 mM) for 6 days. The difference between fluorescence values obtained after 6 days and at Time 0 with an additive was compared to that of BSA incubated without any potential inhibitor. Since some compounds (genistein, kaempferol, naringin, rutin, quercitrin, propyl gallate) were dissolved in dimethylsulfoxide (DMSO) and introduced from a stock DMSO solution, the effect of 1% (final) DMSO on the glycoxidation was checked. The level of glycoxidation found in samples containing DMSO was used as a reference in these cases.

Fluorometric and absorptiometric measurements were done in a Tecan Infinite 200 PRO multimode reader (Tecan Group Ltd., Männedorf, Switzerland).

### 4.8. Statistics

All of the experiments were done at least in triplicate. Data are reported in the form of arithmetic mean values and standard deviations. Differences between means were analyzed using a two-tailed Student’s *t*-test using STATISTICA, version 10 (2010; StatSoft Inc., Tulsa, OK, USA). Spearman’s rank correlation coefficient analysis was employed to estimate the relationships between two compared values, assuming linear dependence.

## 5. Conclusions

The present study confirms the sequence of the rate and intensity of glycoxidation induced by different sugars: ribose > fructose > glucose. Our results point to differences in the extent of inhibition by the same compound of glycoxidation induced by various sugars, pointing out that care should be taken in the extrapolation of the results from one sugar to another. We demonstrate efficient inhibition of glycoxidation by various compounds, of which the best inhibitors were polyphenols, pyridoxine and 1-cyano-4-hydroxycinnamic acid.

## Abbreviations

AGEs: advanced glycation end products
AOPP: advanced oxidation protein products
BSA: bovine serum albumin
EDTA: ethylenediaminetetraacetic acid
DETAPA: diethylenetriaminepentaacetic acid
DMSO: dimethylsulfoxide
NBT: nitroblue tetrazolium
NTA: nitrilotriacetic acid
ROS: reactive oxygen species
